# CREB signalling in bipolar disease (Commentary on Gaspar *et al*.)

**DOI:** 10.1111/ejn.12649

**Published:** 2014-07-06

**Authors:** Aarti Jagannath, Russell G Foster

**Affiliations:** Nuffield Laboratory of Ophthalmology, John Radcliffe Hospital, University of OxfordLevels 5–6, West Wing, Headley Way, Oxford, OX3 9DU, UK

Approximately one in 30 individuals across the world suffers from bipolar disorder (BD). With this prevalence, the pathophysiology of BD represents one of the most important unsolved challenges in neuroscience. Although there appears to be a strong hereditary component to the disorder, genome-wide association studies have failed to identify any obvious underlying developmental or signalling pathways. In this issue, Gaspar *et al*. (2014) report two significant developments in this area. The first is the report of alterations in the cAMP–CREB signalling pathway in BD, and the second is the possibility of a biomarker and assay specific for the condition.

The authors derived fibroblasts from skin biopsy specimens, and used viral luciferase-based reporter systems to investigate the extent to which eight major signalling pathways were activated by specific pharmacological agents. Surprisingly, they found large inter-individual differences in signal pathway transduction in response to the same drug, and these were mirrored by smaller, harder to detect, differences at the transcriptional level. Furthermore, they were able to correlate the degree to which melatonin suppression in response to light occurs in each individual with the strength of the cAMP–CREB signal induced by forskolin in their fibroblasts. They extended these studies to patients with BD, and found a three-fold increase in the cAMP–CREB signalling response in BD patients as compared with age-matched matched controls. This is a particularly interesting finding, given the emerging links between cAMP–CREB signalling and circadian rhythms and their disruption in BD.

Several groups have reported disruption of normal sleep and circadian rhythms in BD, and recently, mechanistic links between the two have emerged. For example, lithium, which is the mainstay pharmacotherapy, has well-documented effects on the circadian clock, and at least two clock mutant mice show a mania-like phenotype. cAMP–CREB signalling plays a fundamental role in regulating the circadian clock, both at the level of clock gene rhythmicity, and by setting the phase of the clock in response to environmental time signals such as light. An upregulated CREB-mediated input pathway in bipolar patients may provide one route by which these disruptions of the circadian clock and other CREB-regulated mechanisms, such as synaptic transmission, are produced. Perhaps most exciting is that this study, alongside other recent reports, suggests that CREB-mediated pathways may provide novel pharmacological targets for the treatment of BD. In addition to a lack of validated targets, drug discovery for BD has been slow, because of the scarcity of both biomarkers and relevant research models that can be used for high-throughput analysis. The authors provide one such assay, the strength of cAMP–CREB signalling in patient-derived cells, which may serve as a valuable tool with which to fight this widespread and debilitating condition.

This study raises many questions and avenues for future work. Do these changes in cAMP–CREB signalling underlie altered neuronal signalling in BD? Do they underlie circadian disruption? Is cAMP–CREB signalling altered in high-risk populations, and can this be used as a predictive tool for the risk of developing disease? Further studies on the fibroblasts from bipolar patients should yield valuable information for addressing such questions.
